# Perioperative management of renin–angiotensin system inhibitors in patients undergoing elective major noncardiac surgery: a mixed model investigation using systematic review, meta-analysis, multicentre service evaluation, and national survey

**DOI:** 10.1016/j.bja.2025.06.026

**Published:** 2025-07-31

**Authors:** Evangelia Giannas, Amour Patel, Priyanthi Dias, Rory J. Heath, Rhona Sinclair, Katy Surman, Amelia Milton, Alexander Middleditch, Tom.E.F. Abbott, Gareth L. Ackland, Rupert M. Pearse, Henrike Janssen, contributors, Ahmed Almeer, Ruth Inez Armstrong, Karen Collins, Mandeep-Kaur Phull, Mark Lowes, Gary Minto, John Paul, Sharon Sarah Thomas, Louise Savic, Victoria White, Valentin Weber

**Affiliations:** 7 Barking Havering and Redbridge University NHS Trust, Barking, UK; 8 University Hospitals Bristol and Weston NHS Foundation Trust, Bristol, UK; 9 University Hospitals Plymouth, Plymouth, UK; 10 Leeds Teaching Hospitals NHS Trust, Leeds, UK; 11 Faculty of Medicine and Dentistry, William Harvey Research Institute, Queen Mary University of London, London, UK; 1 Faculty of Medicine and Dentistry, William Harvey Research Institute, Queen Mary University of London, London, UK; 2 University Hospitals Plymouth, Plymouth, UK; 3 Royal Victoria Infirmary, Newcastle upon Tyne, UK; 4 Barking Havering and Redbridge University NHS Trust, Barking, UK; 5 Leeds Teaching Hospitals NHS Trust, Leeds, UK; 6 Sir Humphry Davy Department of Anaesthesia, University Hospitals Bristol NHS Foundation Trust, Bristol, UK

**Keywords:** elective noncardiac surgery, perioperative hypertension, perioperative hypotension, perioperative mortality, renin–angiotensin system inhibitor

## Abstract

**Background:**

The risks and benefits of stopping or continuing renin–angiotensin system (RAS) inhibitors for major noncardiac surgery remain uncertain. We conducted an updated systematic review, national service evaluation, and clinician survey to inform the design of a large clinical trial of perioperative RAS inhibitor use.

**Methods:**

We searched MEDLINE, CINAHL, ProQuest, Cochrane database, Scopus, and Web of Science from January 2000 to October 2024 for randomised controlled trials (RCT) of perioperative RAS inhibitor use. The primary outcome was a composite of mortality and major cardiovascular events (MACE). Secondary outcomes included acute perioperative hypertension and hypotension. Meta-analysis was performed using random effects models. The I^2^ index was used to quantify heterogeneity. We also conducted a prospective clinical service evaluation and clinician survey to describe current clinical practice in UK.

**Results:**

We identified five RCTs (*n*=2848 patients). Stopping RAS inhibitors was not associated with mortality or MACE (odds ratio [OR] 1.21 [0.60–2.42]; *P*=0.59; I^2^=19%). Stopping RAS inhibitors was associated with acute hypertension (OR 1.90 [1.20–3.02]; *P*=0.007; I^2^=8%) but fewer hypotension events (OR 0.62 [0.42–0.90]; *P*=0.01; I^2^=38%). In a service evaluation of 316 patients in seven hospitals, RAS inhibitors were stopped for 248/316 (79%) patients, with 230/248 (93%) restarting these drugs within 48 h after surgery. In the survey, >80% of clinicians asked patients to stop RAS inhibitors before surgery, for variable reasons concerning risks and benefits.

**Conclusions:**

The optimal approach to perioperative RAS inhibitor use remains uncertain. Although UK clinicians often withhold these drugs, this strategy could cause harm.

**Editor’s key points**

• Recent trials have investigated the impact of stopping renin–angiotensin system (RAS) inhibitors for noncardiac surgery. The findings have not been set in context with previous literature and current clinical practice.
• The authors' mixed model approach showed that clinicians are concerned about RAS inhibitor-induced hypotension even though the duration of hypotensive episodes differs marginally.
• Current studies do not provide detailed data on patients at increased risk (e.g. cardiac failure).

Renin–angiotensin system (RAS) inhibitors, comprising angiotensin-converting enzyme (ACE) inhibitors and angiotensin II receptor blockers (ARBs), are first-line therapy for essential hypertension and heart failure, and also used as interventions of secondary prevention in coronary heart disease, diabetes mellitus, and diabetic nephropathy.^1^, ^2^, ^3^, ^4^

A significant number of patients undergoing major noncardiac surgery are treated chronically with RAS inhibitors, indicated by more than 70% in the clinical trial POISE-3.^5^ Whether RAS inhibitors should be stopped or continued perioperatively remains controversial, as evidenced by variable recommendations in international guidelines.^6^^,^^7^ A taskforce of the European Society of Cardiology, endorsed by the European Society of Anaesthesiology and Intensive Care, rendered data on the perioperative management of RAS inhibitors inconclusive.^8^ Their recommendation includes considering perioperative continuation in patients with stable heart failure and withholding in patients without heart failure. One of the main concerns of perioperative continuation is RAS inhibitor-induced episodes of hypotension, which may promote myocardial or kidney injury.^8^^,^^9^ Conversely, discontinuation of RAS inhibitors increases perioperative episodes of hypertension which also promote myocardial injury.^10^

Two recent RCT in patients undergoing elective noncardiac surgery provide new insights into the impact of perioperative stop or continuation of RAS inhibitiors.^11^^,^^12^ In the SPACE RCT, stopping RAS inhibition was associated with more acute hypertensive adverse events (systolic BP >180 mm Hg),^11^ whereas the Stop-or-Not RCT showed an association of continuation of RAS inhibition with more hypotensive events.^12^

We therefore conducted a systematic literature review and meta-analysis of RCTs with the objective to update a previous analysis.^13^ To put these recent trial results in context, we assessed their potential impact on current clinical practice of perioperative RAS inhibitor management in UK by conducting a prospective clinical service evaluation and survey of clinicians focusing on patients undergoing major elective noncardiac surgery.

## Methods

### Systematic review

#### Protocol and registration

We registered the systematic review prospectively with PROSPERO: CRD42024596893. We followed the Preferred Reporting Items for Systematic Reviews and Meta-Analysis (PRISMA) guidelines for this review. Ethical approval was not required for this study.

#### Search strategy

We searched MEDLINE (PubMed), CINAHL (EBSCO host), ProQuest, Cochrane database, Scopus, and Web of Science from January 2000 to October 2024. Search strategies for all databases are provided in Supplementary material. References of selected articles and published systematic reviews were also searched to identify any further relevant articles meeting inclusion criteria. Original research articles were considered in this study provided they met the following criteria: adult patients (age >18 yr) undergoing elective major noncardiac surgical intervention, defined as in need of in-hospital stay; patients were randomised to stop or continuation of existing preoperative RAS inhibition; quantitative outcomes of all-cause mortality, major cardiovascular events (MACE), hypotension, or hypertension were reported. We excluded non-English articles, review articles, non-research letters, commentaries, animal studies, case reports, nonrandomised trials, retrospective analyses, and full-text articles with insufficient information.

#### Study selection and data extraction

Study selection and data extraction were conducted by two independent researchers (EG, HJ). All studies were screened based on title and abstract, followed by full-text review to identify articles meeting inclusion criteria. The full text of these articles was subsequently reviewed to select papers reporting the predefined outcomes. When there was uncertainty regarding eligibility, a third reviewer was consulted (RP). Data were extracted from selected papers by two independent reviewers to a pre-formatted Excel worksheet (Microsoft, Redmond, WA, USA) (EG, HJ) containing the following characteristics: first author, year, study type, surgery type, outcome(s) reported, and duration of follow-up period. Numbers of events were extracted for dichotomous outcomes (Table 1).

**Table 1:** Main characteristics of studies included in the meta-analysis. ACE, angiotensin-converting enzyme; ARB, angiotensin receptor blocker; C, continued; MACE, major cardiovascular events; RAS, renin–angiotensin system; S, stopped. ∗Whether that dose was scheduled for the morning of surgery or the night before.

Author	Year	Studytype	Type or RAS inhibition	Preoperative stop of RAS inhibition	Surgery	Number of patients	Outcomes relevant to analysis reported	Duration of follow-up
Ackland and colleagues^11^	2024	RCT	ACE and ARB	Defined by pharmacokinetics of each drug (48–24 h)	Noncardiac	C: 130	Hypotension, hypertension, MACE, mortality	Until 72 h after surgery
						S: 124		
Bertrand and colleagues^17^	2001	RCT	ARB	On the day before surgery	Vascular	C: 19	Hypotension, hypertension, MACE, mortality	Until hospital discharge
						S: 18		
Legrand and colleagues^12^	2024	RCT	ACE and ARB	48 h	Noncardiac	C: 1107	Hypotension, hypertension, MACE, mortality	Until 28 days after surgery
						S: 1115		
Rajgopal and colleagues^16^	2014	RCT	ACE and ARB	On the day before surgery	Not specified	C: 30	Hypotension	Until 60 minutes after induction
						S: 30		
Shiffermiller and colleagues^15^	2018	RCT	ACE	Final preoperative dose∗	Noncardiac	C: 138	Hypotension, hypertension, mortality	Until hospital discharge
						S: 137		

#### Outcome measures

The primary outcome measure was composite of all-cause mortality and MACE, which was defined as any combination of myocardial infarction, stroke, and acute heart failure. Definitions of outcome were based on the criteria in each study and are described in Supplementary Table 1. The secondary outcomes were hypotension, hypertension, all-cause mortality, and MACE. Hypotension, hypertension, and MACE were defined according to the criteria used by each study (Supplementary Table 1).

#### Statistical analysis

The quality of each RCT was assessed using the revised Cochrane Collaboration risk of bias tool assessing selection bias, concealment bias, performance bias, detection bias, attrition bias, and reporting bias.^14^ The meta-analysis was conducted using Review Manager (RevMan) software (Version 7.12.0; The Cochrane Collaboration, 2024; available at revman.cochrane.org). Dichotomous data were analysed using odds ratio (OR) with 95% confidence intervals (CIs). The prespecified threshold for statistical significance was *P*<0.05. Between-study heterogeneity was assessed using the I^2^ statistic test using *P*<0.1 as the predefined threshold for statistical significance. We used random effects models for pooled analysis regardless of heterogeneity. The results are presented as forest plots where applicable. Funnel plots assessment for asymmetry was not performed, as only five studies were included.

### Prospective cohort service evaluation

The study was prospectively registered and approved as an observational service evaluation (13658) on December 4, 2023, at NHS Barts Health and registered at participating hospitals and retrospectively registered at *osf.io* (https://doi.org/10.17605/OSF.IO/8JEBT). As the study did not record any patient identifiable data and was registered as a service evaluation, ethical approval was not required. The aim was to assess current management of existing RAS inhibitor prescriptions in patients undergoing major noncardiac surgery. We included patients aged ≥50 yr taking an ACE inhibitor or ARB identified by clinical notes and irrespective of length of prescription who were undergoing major elective noncardiac surgery under general anaesthesia. The primary outcome was RAS inhibition stopped before surgery, which was not further specified. Secondary outcomes were the time point at which ACE inhibitor or ARB were restarted after surgery, stratified into within 12 h, within 12–24 h, 24 h, and ≥48 h after surgery.

#### Data collection and analysis

Data were collected prospectively over a 6-month period from December 2023 until June 2024 for patients who met the service evaluation inclusion criteria. Each site included a predefined number of patients. Data were collected by the clinical teams at sites, coordinated by a site lead. Data were entered for each patient on an Excel spreadsheet and shared with the study team (Supplementary Table 2). Besides perioperative management of RAS inhibitors, data collection included age, sex, ASA classification, relevant risk factors, and type of surgical procedure. All data collected were used for routine clinical care in hospital. There was no additional patient or primary care contact. Patient identifiable information was not part of the data. Submitted data were reviewed for completeness and consistency by authorised users within the study group (EG, HJ). We performed a descriptive analysis presenting frequencies (%) for categorical variables and median (range) for continuous variables. We prespecified stratification by surgical category and by risk factor.

### Clinician survey

The online survey was designed using SurveyMonkey (https://www.smartsurvey.co.uk/s/space2/) and retrospectively registered at osf.io (https://doi.org/10.17605/OSF.IO/8JEBT). The aim of the survey was insight into current management of existing prescriptions of RAS inhibitors. The survey ran from October 15, 2023, to October 29, 2023, and was circulated via e-mail amongst surgeons, anaesthetists, physicians, and pharmacists by the study team (Supplementary Table 3). The survey included questions to perioperative RAS inhibitor management, the likelihood of study participation in the future, and a ranked question with the goal of stopping RAS inhibitors. We performed a descriptive analysis presenting frequencies (%) for categorical variables and median (range) for continuous variables.

## Results

### Systematic review

#### Study selection

We identified 304 citations from our initial search and eight further records by hand search through detailed screening of guidelines and review articles. After removing duplicates, 250 were screened for inclusion criteria, from which we identified six RCTs including patients undergoing major noncardiac surgery.^11^^,^^12^^,^^15^, ^16^, ^17^, ^18^ In one study, cardiovascular morbidity and mortality was not defined; therefore, this study was excluded^18^ (Supplementary Fig. 1).

#### Study characteristics

We found five studies comprising 2848 patients with chronic RAS inhibition (1424 stopped, 1424 continued RAS inhibition) compiled in Table 1. Timing of preoperative RAS inhibition varied between studies, ranging from 48 h to the morning of surgery. The definition of hypotension, hypertension, and MACE also varied widely, as indicated in Supplementary Table 1.

#### Publications bias and study quality

The risk of bias assessment for included studies is depicted in Supplementary Figure 2. As the final analysis included <10 studies,^11^^,^^12^^,^^15^, ^16^, ^17^ we did not formerly assess publication bias as per Cochrane guidelines^19^ but searched for unpublished trials (Supplementary Table 4). NCT04506372 was terminated early owing to slow recruitment after enrolling 342 of estimated 3200 patients. An interim analysis after 59 of estimated 100 enrolled patients in NCT01867047 showed no difference for the specified outcomes and led the investigators to terminate the study. After study completion of NCT01091961, corruption of the randomisation process was revealed.

#### Primary outcome: all-cause mortality and major cardiovascular events

**Fig 1:**
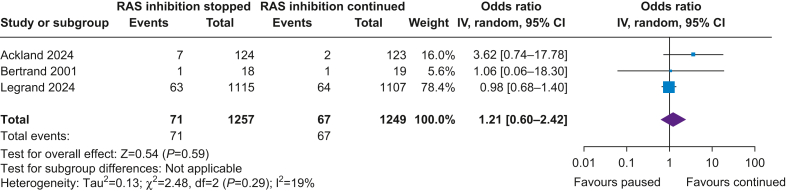
Renin–angiotensin system (RAS) inhibition and composite outcome of major adverse cardiovascular event and mortality. CI, confidence interval; IV, instrumental variable.

Three out of five studies reported all-cause mortality or MACE (Supplementary Table 1), with 1257 patients stopping RAS inhibition and 1249 patients continuing RAS inhibition (Fig. 1).^11^^,^^12^^,^^17^ There was no difference in the composite outcome between patients who stopped or continued RAS inhibitors (OR 1.21; CI [0.60–2.42]; *P*=0.59; I^2^=19%). The omission of either study did not alter the result, albeit changes in heterogeneity (Supplementary Fig. 3).

All-cause mortality was reported in three out of five studies.^11^^,^^12^^,^^15^ There was no difference in all-cause mortality between stopped and continued study arm (OR 0.85; CI [0.39–1.86]; *P*=0.69; I^2^=0%) (Supplementary Fig. 4). No leave-one-out analysis was performed, as only two studies included mortality. Three out of five studies reported MACE as defined in Supplementary Table 1.^11^^,^^12^^,^^17^ There was no difference in the incidence of MACE comparing patients who stopped and continued RAS inhibition (OR 1.51; CI [0.41–5.53]; *P*=0.53; I^2^=35%) (Supplementary Fig. 5). The omission of any study did not alter the results, albeit changes in heterogeneity (Supplementary Fig. 6).

#### Secondary outcomes

##### Acute hypotension

**Fig 2:**
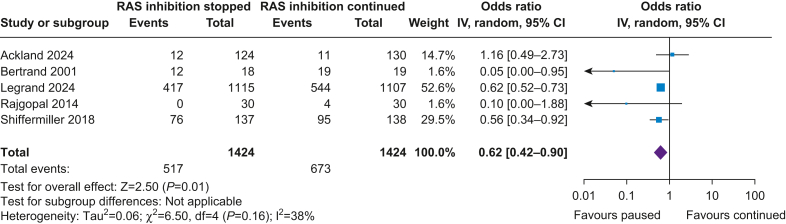
Renin–angiotensin system (RAS) inhibition and acute hypotension. CI, confidence interval; IV, instrumental variable.

Hypotension was defined using highly variable, non-concordant criteria in five trials. The definition of hypotension was either based on systolic BP,^15^^,^^17^ mean arterial pressure,^12^ or the need for clinical intervention with vasopressors^11^^,^^16^ (Supplementary Table 1). The incidence of hypotension was 36.3% in the stopped arm and 47.3% in the continued arm. Stopping RAS inhibitor use was associated with fewer episodes of hypotension, albeit with high heterogeneity (OR 0.62; CI [0.42–0.90]; *P*=0.01; I^2^=38%) (Fig. 2). Leaving-one-out analysis reduced heterogeneity only in the case of omitting the study by Bertrand and colleagues^17^ with favouring the pause of RAS inhibitor (Supplementary Fig. 7).

##### Acute hypertension

**Fig 3:**
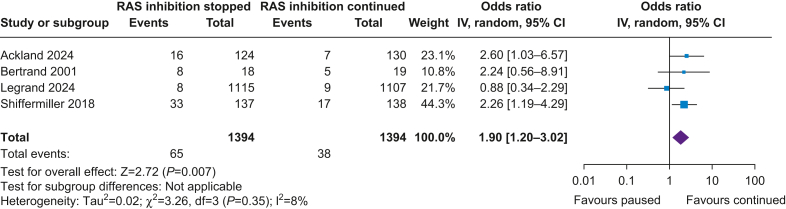
Renin–angiotensin system (RAS) inhibition and acute hypertension. CI, confidence interval; IV, instrumental variable.

Four out of five studies reported hypertension with varying definitions. Most studies based the definition on systolic BP^11^^,^^15^^,^^17^ and one on mean arterial pressure plus need of antihypertensive therapy^12^ (Supplementary Table 1). The incidence of hypertension was 4.7% in the stopped arm and 2.7% in the continued arm. Stopping RAS inhibition was associated with more episodes of hypertension, with moderate heterogeneity (OR 1.90; CI [1.20–3.02]; *P*=0.007; I^2^=8%) (Fig. 3). Leave-one-out analysis reduced heterogeneity when omitting the study by Legrand and colleagues,^12^ with favouring the continuation of RAS inhibitors (Supplementary Fig. 8).

#### GRADE analysis

We performed a Grading of Recommendations, Assessment, Development, and Evaluation Analysis (GRADE) to summarise our findings (Supplementary Fig. 9). Overall, we saw a low risk of bias, but a low or very low certainty of evidence for all outcomes except hypotension where there was a moderate certainty of evidence.

### National prospective service evaluation

#### Patient characteristics

We received data for 370 patients from seven participating hospitals. After reviewing the data for inclusion criteria, 316 patients were analysed (Supplementary Table 5). Reasons for exclusions are specified in Supplementary Figure 10.

#### Perioperative management of renin–angiotensin system inhibition

From the 210 patients receiving ACE inhibitors, 160 (76.2%) were advised to stop medication before surgery compared with 50 (23.8%) advised to continue. For patients taking ARB, 89 of 107 patients (83.2%) were advised to stop RAS inhibition compared with 18 (16.8%) continuing as per their prescription (Table 2). Most patients restarted ACE inhibition within 48 h (91.2%) (Table 2). The findings were consistent when patients were stratified by surgical subspecialty or risk factor (Supplementary Tables 6 and 7).

**Table 2:** Perioperative ACE inhibitor and ARB management from national service evaluation. ACE, angiotensin-converting enzyme; ARB, angiotensin receptor blocker. ∗One patient received both ACE inhibitor and ARB.

Preoperative RAS inhibitor management	Patients with ACE inhibitorNumber of patients with available data, *n* (%) *N*=210 (100)		Patients with ARBNumber of patients with available data, *n* (%) *N*=107 (100)		Patients with any RAS inhibitorNumber of patients with available data, *n* (%) *N*=316 (100)	
	Stopped	Continued	Stopped	Continued	Stopped	Continued
	160 (76.2)	50 (23.8)	89 (83.2)	18 (16.8)	248 (78.5)∗	68 (21.2)

**Timing of postoperatively restarted RAS inhibitor**	**Patients with ACE inhibitorNumber of patients with available data, *n* (%) *N*=160 (100)**		**Patients with ARBNumber of patients with available data, *n* (%) *N*=89 (100)**		**Patients with any RAS inhibitorNumber of patients with available data, *n* (%) *N*=248 (100)**	

Within 12 h	5 (3.1)		1 (1.1)		6 (2.4)	
Within 12–24 h	61 (38.1)		33 (37.1)		94 (37.9)	
Within 24–48 h	24 (15.0)		10 (11.2)		34 (13.7)	
From 48 h until discharge	56 (35.0)		41 (46.1)		96 (38.7)∗	
Not restarted until discharge	14 (8.8)		4 (4.5)		18 (7.3)	

### Clinician survey

The survey was completed by 247 respondents, of whom 4.5% were surgeons and 92.3% anaesthetists. Most clinicians advised patients to stop RAS inhibition before surgery (Table 3). The majority (64.8%) ranked avoidance of hypotension as their most important goal of stopping RAS inhibitors, followed by the avoidance of high BP (18.7%).

**Table 3:** Perioperative ACE inhibitor and ARB management from clinician survey. ACE, angiotensin-converting enzyme; ARB, angiotensin receptor blocker; NIHR, National Institute for Health and Care Research.

Question	Answer options	Number of clinicians with available data, *n* (%) *N*=247 (100)
What advice do you give patients taking ACE inhibitors who are preparing for major noncardiac surgery?	Continue ACE inhibitor	34 (13.8)
	Stop 2 or more days before surgery	13 (5.3)
	Stop 1 day before surgery	35 (14.2)
	Stop on the day of surgery itself	154 (62.4)
	Stop but duration varies with the patient and drug	11 (4.5)
If you did stop ACE inhibitors, when would you normally restart them after major surgery?	Within 12 h after surgery	20 (8.1)
	12–24 h after surgery	97 (39.3)
	24 h after surgery	86 (34.8)
	48 h or more after surgery	44 (17.8)
What advice do you give patients taking ARBs who are preparing for major noncardiac surgery?	Continue ARB	42 (17.0)
	Stop 2 or more days before surgery	14 (5.7)
	Stop 1 day before surgery	37 (15.0)
	Stop on the day of surgery itself	144 (58.3)
	Stop but duration varies with the patient and drug	10 (4.1)
If you did stop ARBs, when would you normally restart them after major surgery?	Within 12 h after surgery	24 (9.7)
	12–24 h after surgery	93 (37.7)
	24 h after surgery	89 (36.0)
	48 h or more after surgery	41 (16.6)
Does your hospital have routine guidance on stopping or continuing ACE inhibitors and ARBs before major noncardiac surgery?	We have no locally agreed guideline	60 (24.2)
	We have a guideline, but we only use this occasionally	18 (7.3)
	We have a guideline we use in most or all patients	169 (68.4)
If NIHR funded a major trial of stopping *vs* continuing ACE inhibitors and ARBs before major noncardiac surgery, how likely would you be to support randomising most of your patients? We recognise this will not be appropriate for every patient.	Very likely	122 (49.4)
	Likely	87 (35.2)
	Neither likely nor unlikely	24 (9.7)
	Unlikely	9 (3.64)
	Very unlikely	5 (2.0)
If NIHR funded a major trial of stopping *vs* continuing ACE inhibitors and ARBs before surgery, would your hospital be likely to take part?	Very likely	100 (40.5)
	Likely	106 (42.9)
	Neither likely nor unlikely	35 (14.2)
	Unlikely	5 (2.0)
	Very unlikely	1 (0.4)

## Discussion

Our meta-analysis of five RCTs including 2848 patients showed that pausing RAS inhibition does not increase the risk for the composite outcome of MACE and death. However, stopping RAS inhibition was associated with acute hypertension, in contrast to continuation being associated with acute hypotension. Our service evaluation and clinicians’ survey highlighted that stopping RAS inhibition in the perioperative period is common, irrespective of surgical subspecialty and comorbidities, and driven by the perception that stopping helps to avoid hypotension.

Our meta-analysis showed an association of continuing RAS inhibition and acute hypotension, which is associated with cardiovascular complications and mortality after noncardiac surgery.^10^^,^^20^^,^^21^ However, the definition of hypotension varied widely, reducing the generalisation of this finding. The omission of the study by either Legrand and colleagues^12^ or Shiffermiller and colleagues^15^ led to a nonsignificant result, albeit increasing heterogeneity. Additionally, our analysis does not take the length of hypotensive episodes in account as few studies provided information. Shiffermiller and colleagues^15^ report a median length of hypotensive episodes of 2 min (RAS inhibition stopped) compared with 7 min (RAS inhibition continued) and Legrand and colleagues^12^ report 6 min (RAS inhibition stopped) compared with 9 min (RAS inhibition continued). Although this difference is statistically significant, it is implausible that this finding is clinically relevant.^22^^,^^23^ Hypotensive episodes therefore still occur in patients who stop RAS inhibition and episodes are only marginally longer in patients who continue. Our meta-analysis showed an association between the stop of RAS inhibitors and perioperative hypertension with wide variation of definition. Hypertensive episodes are associated with myocardial injury in the perioperative phase underlining bidirectional swings in BP as harmful.^10^ The omission of any study besides Legrand and colleagues^12^ rendered the result nonsignificant while increasing heterogeneity. Surgical and perioperative care in the study by Bertrand and colleagues^17^ conducted in 2001 differs from other included studies, but its omission does not drive a unique effect.

Our national service evaluation and clinician survey showed that stopping RAS inhibition is common, independently of procedure and comorbidities. Clinicians’ main goal for stopping RAS inhibition was avoidance of hypotension, indicating that clinicians’ worry about induction of hypotension outweighs that of hypertension. It may also indicate that fewer clinicians are aware of the association of stopping RAS inhibitors and acute hypertension. Conversely, the risk of stroke as a potential complication of hypertensive episodes is a source of major concern for patients undergoing surgery.

The main limitations of our systematic review are the heterogenous definitions of hypotension and hypertension and short-term follow-up. POISE-3, a large international trial of a complex intervention to prevent perioperative hypotension is limited by only 57% of patients receiving their RAS inhibitor strategy as intended.^5^^,^^24^ POISE-3 was therefore not included in our analysis. We did not assess publication bias, as we included fewer than 10 studies. Three relevant registered trials were not published. One trial was terminated early for poor recruitment, whereas another identified compromised randomisation after conclusion, highlighting difficulties of performing large at-scale trials. Our service evaluation and clinician survey were limited geographically and may not reflect practice throughout the UK and further afield. The service evaluation included data from a 6-month period that only reflects recent selected practice.

Current studies did not allow us to make conclusions regarding other specific perioperative complications such as myocardial injury, a recognised complication after noncardiac surgery and strongly associated with both in-hospital and post-discharge mortality.^25^ In the SPACE RCT, stopping RAS inhibitors did not lead to a higher incidence of myocardial injury.^11^ However, a *post hoc* analysis of SPACE stratifying patients for risk of cardiovascular events, assessed by preoperative NT-proBNP, highlighted the potential impact of stopping RAS inhibitors in subgroups at higher risk of complications.^26^ Patients with preoperative low NT-proBNP plasma concentrations experienced myocardial injury more frequently when RAS inhibition was stopped, compared with those who continued.^26^ This finding may be accounted for by rebound hypertension^27^ or modification of inflammatory pathways,^26^ highlighting that patients with heart failure may respond differently when RAS inhibitors are stopped abruptly. No clinical trial has specifically focused on the management of RAS inhibitors in patients with heart failure, who are at higher perioperative risk of adverse outcomes compared with patients with coronary heart disease alone.^28^ Retrospective data suggests that stopping RAS inhibition increases mortality and hospital readmission.^29^^,^^30^ In the study by Legrand and colleagues,^12^ 6% of patients were diagnosed with heart failure, comparable with <10% in the SPACE RCT.^11^ However, nearly half of preoperative diagnosis of heart failure is missed in patients undergoing noncardiac surgery.^31^ An *a priori* analysis of NT-proBNP plasma concentrations in the SPACE RCT^11^ indicated likely cardiovascular dysfunction in 42% of the study population, despite only 6.7% of patients formally diagnosed with heart failure.^26^ In our service evaluation, 64.5% patients with diagnosed heart failure were advised to stop RAS inhibitors, highlighting that even in a group at high perioperative risk stopping RAS inhibitors is common. Acute kidney injury was only assessed in the studies by Ackland and colleagues^11^ and Legrand^27^ with no detected relationship of continuation of RAS inhibition and acute kidney injury.

In comparison with a recently published meta-analysis focusing on the perioperative management of RAS inhibitors,^32^ we highlight that our study provides a critical interpretation of the potentially clinically irrelevant increase of hypotensive episodes owing to short length, and further interpretation pertaining to hypertensive events and patient groups at elevated risk.

In conclusion, we present an updated meta-analysis of the impact of perioperative stopping RAS inhibition and current practice in UK. These studies suggest strongly that many aspects of perioperative RAS inhibition remain unclear, including the impact of their management on myocardial injury and higher-risk groups such as patients with heart failure.

## Authors’ contributions

Study design, data collection, interpretation of results, and manuscript preparation: EG, AP, PD, RH, RS, KS, AmM, AlM, TA, GA, RP, HJ

## Funding

John Snow Anaesthesia Intercalated Award 2024 (to EG); BJA Centenary Grant (2024–7) (to HJ); NIHR Development & Skills Enhancement award (ref. 305701 to TA); UK National Institute for Academic Anaesthesia (British Oxygen Company research chair grant to GLA); NIHR Advanced Fellowship (NIHR300097 to GLA); and British Heart Foundation Program grants (RG/F/24/110146, RG/14/4/30736; RG/19/5/34463 to GLA).

## Declarations of interest

HJ was an Editorial Fellow for the *British Journal of Anaesthesia*. TA has received research funding from NIHR, Barts Charity, the Academy of Medical Sciences, The Royal College of Anaesthetists, and British Journal of Anaesthesia (BJA); has received honoraria from MSD and Edwards Life Sciences; and is Social Media Editor for the *British Journal of Anaesthesia* and *BJA* *Open*. GLA is an editor of the *British Journal of Anaesthesia*.

## References

[1] McEvoy J.W., McCarthy C.P., Bruno R.M. (2024). 2024 ESC Guidelines for the management of elevated blood pressure and hypertension. Eur Heart J.

[2] McDonagh T.A., Metra M., Authors/Task Force Members (2024). 2023 focused update of the 2021 ESC guidelines for the diagnosis and treatment of acute and chronic heart failure: Developed by the task force for the diagnosis and treatment of acute and chronic heart failure of the European Society of Cardiology (ESC) with the special contribution of the Heart Failure Association (HFA) of the ESC. Eur J Heart Fail.

[3] Wright J.T., Williamson J.D., The SPRINT Research Group (2015). A randomized trial of intensive versus standard blood-pressure control. N Engl J Med.

[4] Zhang W., Zhang S., Deng Y. (2021). Trial of intensive blood-pressure control in older patients with hypertension. N Engl J Med.

[5] Marcucci M., Painter T.W., Conen D. (2023). Hypotension-avoidance versus hypertension-avoidance strategies in noncardiac surgery: an international randomized controlled trial. Ann Intern Med.

[6] Fleisher L.A., Fleischmann K.E., Auerbach A.D. (2014). ACC/AHA guideline on perioperative cardiovascular evaluation and management of patients undergoing noncardiac surgery: a report of the American College of Cardiology/American Heart Association Task Force on Practice Guidelines. Circulation.

[7] Kristensen S.D., Knuuti J., Saraste A. (2014). ESC/ESA guidelines on non-cardiac surgery: cardiovascular assessment and management: the Joint Task Force on non-cardiac surgery: cardiovascular assessment and management of the European Society of Cardiology (ESC) and the European Society of Anaesthesiology (ESA). Eur Heart J.

[8] Halvorsen S., Mehilli J., Cassese S. (2022). ESC Guidelines on cardiovascular assessment and management of patients undergoing non-cardiac surgery. Eur Heart J.

[9] Ackland G.L., Abbott T.E.F. (2022). Hypotension as a marker or mediator of perioperative organ injury: a narrative review. Br J Anaesth.

[10] Abbott T.E.F., Pearse R.M., Archbold R.A. (2018). A prospective international multicentre cohort study of intraoperative heart rate and systolic blood pressure and myocardial injury after noncardiac surgery: results of the VISION study. Anesth Analg.

[11] Ackland G.L., Patel A., Abbott T.E.F. (2024). Discontinuation vs. continuation of renin–angiotensin system inhibition before non-cardiac surgery: the SPACE trial. Eur Heart J.

[12] Legrand M., Falcone J., Cholley B. (2024). Continuation vs discontinuation of renin-angiotensin system inhibitors before major noncardiac surgery: the stop-or-not randomized clinical trial. JAMA.

[13] Hollmann C., Fernandes N.L., Biccard B.M. (2018). A systematic review of outcomes associated with withholding or continuing angiotensin-converting enzyme inhibitors and angiotensin receptor blockers before noncardiac surgery. Anesth Analg.

[14] Higgins J.P.T., Altman D.G., Gotzsche P.C. (2011). The Cochrane Collaboration’s tool for assessing risk of bias in randomised trials. BMJ.

[15] Shiffermiller J.F., Monson B.J., Vokoun C.W. (2018). Prospective Randomized Evaluation of Preoperative Angiotensin-Converting Enzyme Inhibition (PREOP-ACEI). J Hosp Med.

[16] Rajgopal R., Rajan S., Sapru K., Paul J. (2014). Effect of pre-operative discontinuation of angiotensin-converting enzyme inhibitors or angiotensin II receptor antagonists on intra-operative arterial pressures after induction of general anesthesia. Anesth Essays Res.

[17] Bertrand M., Godet G., Meersschaert K., Brun L., Salcedo E., Coriat P. (2001). Should the angiotensin II antagonists be discontinued before surgery?. Anesth Analg.

[18] Yuan R., Xu M., Hu C. (2024). Hemodynamic effects of withholding vs. continuing angiotensin II receptor blockers on the day of prone positioning spinal surgery in elderly patients. Front Med.

[19] Boutron I., Page M.J., Higgins J.P.T., Altman D.G., Lundh A., Hróbjartsson A., Higgins J.P.T., Thomas J., Chandler J., Cumpston M., Li T., Page M.J., Welch V.A. (2024). Cochrane Handbook for Systematic Reviews of Interventions version 6.5. Cochrane.

[20] Van Waes J.A.R., Van Klei W.A., Wijeysundera D.N., Van Wolfswinkel L., Lindsay T.F., Beattie W.S. (2016). Association between intraoperative hypotension and myocardial injury after vascular surgery. Anesthesiology.

[21] Monk T.G., Bronsert M.R., Henderson W.G. (2015). Association between intraoperative hypotension and hypertension and 30-day postoperative mortality in noncardiac surgery. Anesthesiology.

[22] Walsh M., Devereaux P.J., Garg A.X. (2013). Relationship between intraoperative mean arterial pressure and clinical outcomes after noncardiac surgery. Anesthesiology.

[23] Liem V.G.B., Hoeks S.E., Mol K.H.J.M. (2020). Postoperative hypotension after noncardiac surgery and the association with myocardial injury. Anesthesiology.

[24] Devereaux P.J., Marcucci M., Painter T.W. (2022). Tranexamic acid in patients undergoing noncardiac surgery. N Engl J Med.

[25] Devereaux P.J., Biccard B.M., Writing Committee for the VISION Study Investigators (2017). Association of postoperative high-sensitivity troponin levels with myocardial injury and 30-day mortality among patients undergoing noncardiac surgery. JAMA.

[26] Gutierrez Del Arroyo A., Patel A., Abbott T.E.F. (2024). Preoperative N-terminal pro-B-type natriuretic peptide and myocardial injury after stopping or continuing renin–angiotensin system inhibitors in noncardiac surgery: a prespecified analysis of a phase 2 randomised controlled multicentre trial. Br J Anaesth.

[27] Legrand M. (2024). Should renin–angiotensin system inhibitors be held prior to major surgery?. Br J Anaesth.

[28] Van Diepen S., Bakal J.A., McAlister F.A., Ezekowitz J.A. (2011). Mortality and readmission of patients with heart failure, atrial fibrillation, or coronary artery disease undergoing noncardiac surgery: an analysis of 38 047 patients. Circulation.

[29] Gilstrap L.G., Fonarow G.C., Desai A.S. (2017). Initiation, continuation, or withdrawal of angiotensin-converting enzyme inhibitors/angiotensin receptor blockers and outcomes in patients hospitalized with heart failure with reduced ejection fraction. J Am Heart Assoc.

[30] Lee S.M., Takemoto S., Wallace A.W. (2015). Association between withholding angiotensin receptor blockers in the early postoperative period and 30-day mortality. Anesthesiology.

[31] Golbus J.R., Joo H., Janda A.M. (2022). Preoperative clinical diagnostic accuracy of heart failure among patients undergoing major noncardiac surgery: a single-centre prospective observational analysis. BJA Open.

[32] Ahmed M., Fatima E., Shafiq A. (2024). Continuation versus discontinuation of renin-angiotensin aldosterone system inhibitors before non-cardiac surgery: a systematic review and meta-analysis. J Clin Anesth.

