# Supplementary material for: Perioperative management of renin–angiotensin system inhibitors in patients undergoing elective major noncardiac surgery: a mixed model investigation using systematic review, meta-analysis, multicentre service evaluation, and national survey
Source: Br J Anaesth. 2025 Jul 31;135(4):861–9. doi: 10.1016/j.bja.2025.06.026 (PMC12674026; doi:10.1016/j.bja.2025.06.026)
Supplement: Multimedia component 1 [file mmc1.docx]

**Supplementary material**

Search strategies for systematic literature review

**PubMed (187 results)**

(((((((operative OR surgical OR surgery OR "Surgical Procedures, Operative"[Mesh]))) AND ((((((((Angiotensin II Receptor Blockers[Title/Abstract]) OR Angiotensin Receptor Blockers[Title/Abstract]) OR Angiotensin II Receptor Antagonists[Title/Abstract]) OR Angiotensin Receptor Antagonists[Title/Abstract]) OR (("Angiotensin Receptor Antagonists"[Mesh]) AND "Angiotensin II Type 1 Receptor Blockers"[Mesh])))) OR ((((((("Angiotensin-Converting Enzyme Inhibitors"[Mesh]) OR angiotensin-converting enzyme inhibitors[Title/Abstract]) OR Angiotensin Converting Enzyme Inhibitors[Title/Abstract]) OR Angiotensin-Converting Enzyme Antagonists[Title/Abstract]) OR ACE Inhibitors[Title/Abstract]) OR ACE-I[Title/Abstract]))))) NOT (Cardiac surgical procedures OR heart surgery OR cardiac surgery OR "Cardiac Surgical Procedures"[Mesh]))) AND ((((((((((discontinuing) OR omission) OR discontinue) OR cease OR stop OR withhold) OR stopping) OR Withholding)) OR "Withholding Treatment"[Mesh])AND (("2000/01/01"[Date - MeSH]: "2024"[Date - MeSH]))

**Cochrane (11 results)**

#1 ("operative"):ti,ab,kw OR ("surgical"):ti,ab,kw OR ("surgery"):ti,ab,kw OR (Surgical procedure):ti,ab,kw

#2 ("angiotensin ii receptor blockers"):ti,ab,kw OR ("Angiotensin Receptor Blockers"):ti,ab,kw OR ("Angiotensin II Receptor Antagonists"):ti,ab,kw OR ("Angiotensin Receptor Antagonists"):ti,ab,kw OR ("Angiotensin Receptor Antagonists"):ti,ab,kw

#3 ("Angiotensin II Type 1 Receptor Blockers"):ti,ab,kw OR ("Angiotensin-Converting Enzyme Inhibitors"):ti,ab,kw OR ("Angiotensin-Converting Enzyme Antagonists"):ti,ab,kw OR ("ACE Inhibitors"):ti,ab,kw OR ("ACE-I"):ti,ab,kw

#4 ("Cardiac surgical procedures"):ti,ab,kw OR ("heart surgery"):ti,ab,kw OR ("cardiac surgery"):ti,ab,kw OR ("Cardiac Surgical Procedures"):ti,ab,kw

#5 ("discontinuing"):ti,ab,kw OR ("omission"):ti,ab,kw OR ("discontinue"):ti,ab,kw OR ("cease "):ti,ab,kw OR ("stop"):ti,ab,kw OR ("withhold"):ti,ab,kw OR ("Withholding"):ti,ab,kw OR ("Withholding treatment"):ti,ab,kw

#6 #1 AND #2 AND #3 AND #5 NOT #4 with Cochrane Library publication date Between Jan 2000 and Feb 202

**ProQuest (30 results)**

Set#: S1

Searched for: noft(angiotensin ii receptor blockers) OR noft(angiotensin receptor blockers) OR noft(angiotensin ii receptor antagonists) OR noft(angiotensin receptor antagonists) OR noft(Angiotensin Receptor Antagonists)

Databases: Coronavirus Research Database, Health Research Premium Collection, MEDLINE®

Results: 55513

Set#: S2

Searched for: noft(operative) OR noft(surgical procedure) OR noft(surgical) OR noft(Surgery)

Databases: APA PsycArticles®, APA PsycInfo®, British Nursing Index, Coronavirus Research Database, Health Research Premium Collection, MEDLINE®, PTSDpubs, Publicly Available Content Database

Results: 6114080

Set#: S3

Searched for: noft(Angiotensin II Type 1 Receptor Blockers) OR noft(Angiotensin-Converting Enzyme Inhibitors) OR noft(Angiotensin-Converting Enzyme Antagonists) OR noft(ace-inhibitors) OR noft(ace-i)

Databases: APA PsycArticles®, APA PsycInfo®, British Nursing Index, Coronavirus Research Database, Health Research Premium Collection, MEDLINE®, PTSDpubs, Publicly Available Content Database

Results: 88667

Set#: S4

Searched for: noft(Discontinuing) OR noft(Omission) OR noft(Discontinue) OR noft( cease) OR noft( stop) OR noft(withhold) OR noft( stopping) OR noft(Withholding) OR noft(withholding treatment)

Databases: APA PsycArticles®, APA PsycInfo®, British Nursing Index, Coronavirus Research Database, Health Research Premium Collection, MEDLINE®, PTSDpubs, Publicly Available Content Database

Results: 459536

Set#: S5

Searched for: noft(Cardiac Surgical Procedures) OR noft(heart surgery) OR noft(Cardiac Surgery) OR noft(Cardiac Surgical Procedures)

Databases: APA PsycArticles®, APA PsycInfo®, British Nursing Index, Coronavirus Research Database, Health Research Premium Collection, MEDLINE®, PTSDpubs, Publicly Available Content Database

Results: 562943

Set#: S6

Searched for: [S1] AND [S2] AND [S3] AND [S4] NOT [S5]

Databases: APA PsycArticles®, APA PsycInfo®, British Nursing Index, Coronavirus Research Database, Health Research Premium Collection, MEDLINE®, PTSDpubs, Publicly Available Content Database

**Scopus (23 results)**

TITLE-ABS ( "Angiotensin ii receptor blockers" OR "angiotensin receptor blockers" OR "angiotensin ii receptor antagonists" OR "angiotensin receptor antagonists" AND operative OR "surgical procedure" OR surgical OR surgery AND "Angiotensin II Type 1 Receptor Blockers" OR "Angiotensin-Converting Enzyme Inhibitors" OR "Angiotensin-Converting Enzyme Antagonists" OR ace-inhibitors OR ace-i AND discontinuing OR omission OR discontinue OR cease OR stop OR withhold OR stopping OR withholding OR "withholding treatment" AND NOT "heart surgery" OR "cardiac surgery" OR "Cardiac Surgical Procedures" ) AND PUBYEAR > 2000 AND PUBYEAR < 2025 AND PUBYEAR > 2000 AND PUBYEAR < 2025

**Web of Science (38 results)**

(((((ALL=("Angiotensin ii receptor blockers" OR "angiotensin receptor blockers" OR "angiotensin ii receptor antagonists" OR "angiotensin receptor antagonists")) AND ALL=(operative OR "surgical procedure" OR surgical OR surgery)) AND ALL=("Angiotensin II Type 1 Receptor Blockers" OR "Angiotensin-Converting Enzyme Inhibitors" OR "Angiotensin-Converting Enzyme Antagonists" OR ace-inhibitors OR ace-i )) AND ALL=(discontinuing OR omission OR discontinue OR cease OR stop OR withhold OR stopping OR withholding OR "withholding treatment" ))) NOT ALL=("heart surgery" OR "cardiac surgery" OR "Cardiac Surgical Procedures")

**EBSCO (7 results)**

| S6 | S1 AND S2 AND S3 AND S4 NOT S5 | Limiters - Publication Date: 20000101-20240331 Search modes - Find any of my search terms | Interface - EBSCOhost Research Databases Search Screen - Advanced Search Database - CINAHL | 7 |
| --- | --- | --- | --- | --- |
| S5 | AB Cardiac Surgical Procedures OR AB heart surgery OR AB Cardiac Surgery OR AB Cardiac Surgical Procedures OR TI Cardiac Surgical Procedures OR TI heart surgery OR TI Cardiac Surgery OR TI Cardiac Surgical Procedures | Limiters - Publication Date: 20000101-20240331 Search modes - Boolean/Phrase | Interface - EBSCOhost Research Databases Search Screen - Advanced Search Database - CINAHL | 15,008 |
| S4 | AB discontinuing OR AB Omission OR AB discontinue OR AB cease OR AB stop OR AB withhold OR AB stopping OR AB Withholding OR AB withholding treatment OR TI discontinuing OR TI Omission OR TI discontinue OR TI cease OR AB stop OR TI withhold OR TI stopping OR TI Withholding OR TI withholding treatment | Limiters - Publication Date: 20000101-20240331 Search modes - Boolean/Phrase | Interface - EBSCOhost Research Databases Search Screen - Advanced Search Database - CINAHL | 30,345 |
| S3 | AB operative OR AB surgical procedure OR AB surgical OR AB surgery OR TI operative OR TI surgical procedure OR TI surgical OR TI surgery | Limiters - Publication Date: 20000101-20240331 Search modes - Boolean/Phrase | Interface - EBSCOhost Research Databases Search Screen - Advanced Search Database - CINAHL | 445,376 |
| S2 | AB Angiotensin II Type 1 Receptor Blockers OR AB Angiotensin-Converting Enzyme Inhibitors OR AB Angiotensin-Converting Enzyme Antagonists OR AB ace-inhibitors OR AB ace-i OR TI Angiotensin II Type 1 Receptor Blockers OR TI Angiotensin-Converting Enzyme Inhibitors OR TI Angiotensin-Converting Enzyme Antagonists OR TI ace-inhibitors OR TI ace-i | Limiters - Publication Date: 20000101-20240331 Search modes - Boolean/Phrase | Interface - EBSCOhost Research Databases Search Screen - Advanced Search Database - CINAHL | 7,107 |
| S1 | AB angiotensin ii receptor blockers OR AB angiotensin receptor blockers OR AB angiotensin ii receptor antagonists OR AB angiotensin receptor antagonists OR TI angiotensin ii receptor blockers OR TI angiotensin receptor blockers OR TI angiotensin ii receptor antagonists OR TI angiotensin receptor antagonists | Limiters - Publication Date: 20000101-20240331 Search modes - Boolean/Phrase | Interface - EBSCOhost Research Databases Search Screen - Advanced Search Database - CINAHL | 3,981 |

**Supplementary Figure 1 PRISMA flow diagram showing literature research results**. Five studies were used for meta-analysis. PRISMA (Preferred Reporting Items for Systematic Reviews and Meta-Analysis).


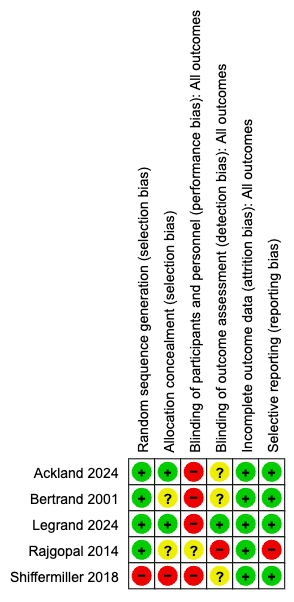


**Supplementary Figure 2 Risk of bias summary**. Five randomised controlled trials were used for meta-analysis and assessed for bias with the revised Cochrane Collaboration risk of bias tool.

**Supplementary Figure 3 Leave-one-out analysis for composite outcome of major cardiovascular event and mortality.** OR, odds ratio; CI, confidence interval.


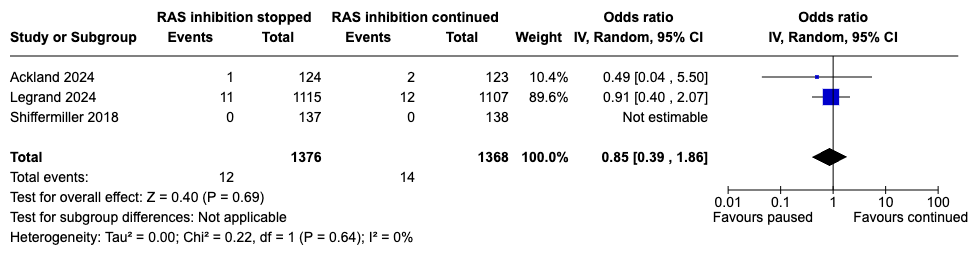


**Supplementary Figure 4 Renin-angiotensin system inhibition and mortality.** RAS, Renin-angiotensin system; IV, instrumental variable; CI, confidence interval.


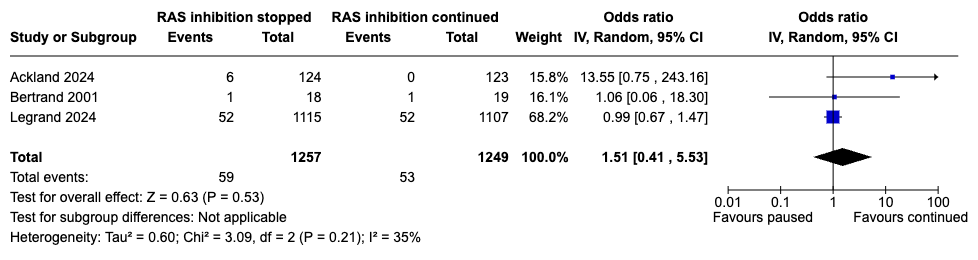


**Supplementary Figure 5 Renin-angiotensin system inhibition and major cardiovascular events.** RAS, Renin-angiotensin system; IV, instrumental variable; CI, confidence interval.

**Supplementary Figure 6 Leave-one-out analysis for major cardiovascular adverse events.** OR, odds ratio; CI, confidence interval.

**Supplementary Figure 7 Leave-one-out analysis for hypotension.** OR, odds ratio; CI, confidence interval.

**Supplementary Figure 8 Leave-one-out analysis for hypertension.** OR, odds ratio; CI, confidence interval.

**Supplementary Figure 9 GRADE analysis. RAS;** Renin-angiotensin system, CI; confidence interval, RCT; randomised controlled trial, OR; Odds Ratio.


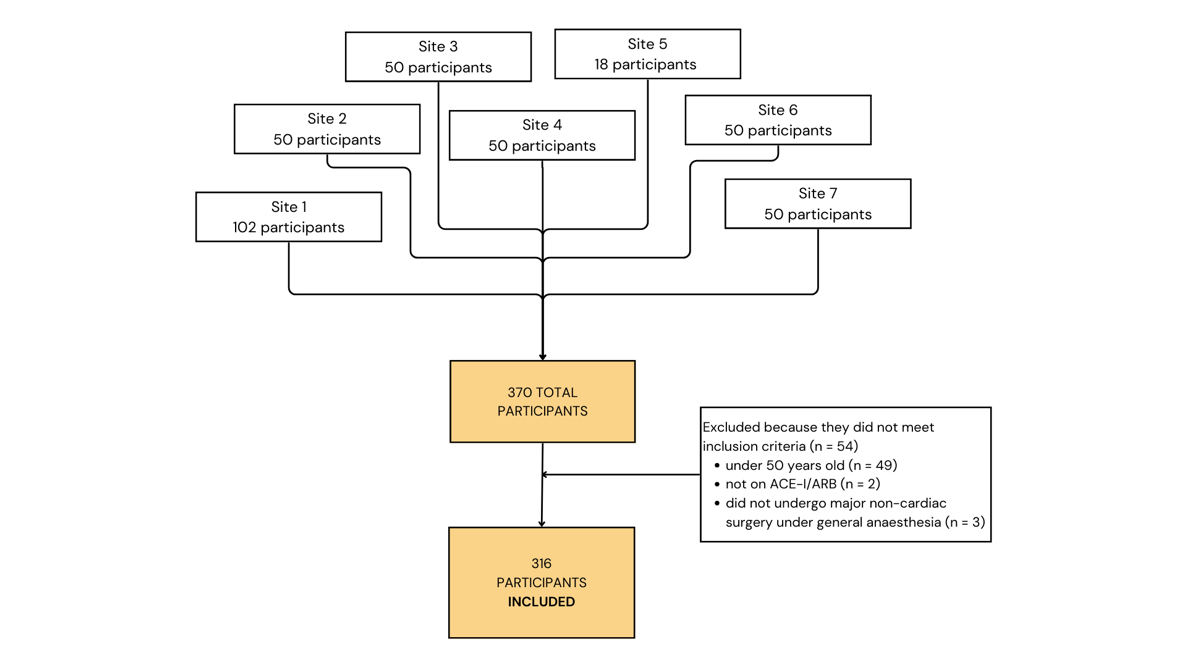


**Supplementary Figure 10 Patient selection in national service evaluation.** Reasons for exclusion are specified.

| **Author** | **Definition of hypotension** | **Definition of hypertension** | **Diagnosis qualifying as MACE** | **Definition of mortality** |
| --- | --- | --- | --- | --- |
| Ackland et al. | Hypotension treated by clinician with vasoactive infusion requiring central venous access from induction to 48 h | SBP >180 mmHg and/or DBP >100 mmHg, from the time of randomization until 48 h after surgery | Myocardial infarction, acute heart failure, stroke within 30 days of surgery^1^ | Death within 30 days of randomisation |
| Bertrand et al. | SBP < 80 mmHg for more than one minute from induction | SBP > 160 mm Hg lasting more than 1 min | ST-abnormalities^2^ |  |
| Legrand et al. | MAP < 60 mmHg or required treatment with vasopressor | Episode of hypertension with MAP ≥ 130 mmHg requiring intravenous  antihypertensive treatment | Compound outcome of postoperative cardiovascular events^3^ | Any cause of death within 28 days following surgery. |
| Rajgopal et al. | Ephedrine received to maintain MAP > 60 mmHg | NA | NA |  |
| Shiffermiller et al. | SBP < 80 mmHg from induction to transfer to PACU | any SBP > 180 mmHg occurring after arrival in the PACU | NA | Not further defined |

**Supplementary Table 1: Outcome definitions of studies included in meta-analysis. MACE, major adverse cardiovascular event;** SBP, Systolic blood pressure; MAP, mean arterial pressure; DBP, diastolic blood pressure; NA, not applicable; PACU postoperative care unit.

**^1^ Myocardial Infarction:** Acute myocardial injury with clinical evidence of acute myocardial ischaemia and with detection of an increase or decrease in cTn values with at least one value above the 99th percentile URL and at least one of the following:

(i) Symptoms of myocardial ischaemia

(ii) New ischaemic ECG changes

(iii) Development of pathological Q waves

(iv) Imaging evidence of new loss of viable myocardium or new regional wall motion abnormality in a pattern consistent with an ischaemic aetiology

(v) Identification of a coronary thrombus by angiography or autopsy.

**^1^ Acute heart failure:** Cardiogenic pulmonary oedema is defined as radiographic or imaging evidence of fluid accumulation in the alveoli due to poor cardiac function, and/or clinical treatment instituted on basis of clinically suspected heart failure.

**^1^** **Stroke:** Defined as a new focal neurological deficit thought to be vascular in origin with signs or symptoms lasting more than 24 hours or leading to death.

**^2^ ST-abnormalities:** ST-abnormalities are not further described within the study

**^3^ Compound outcome of postoperative cardiovascular events**

*(i) acute myocardial infarction:* Occurrence of a de novo myocardial infarction postoperatively based on international criteria (biological, clinical, electrical, and imaging criteria based on European Heart Journal (2012) 33, 2551‐2567)

*(ii) arterial or venous thrombosis:* Occurrence of an acute complete vascular occlusion within 28 days post‐surgery authenticated by imaging (ultrasound, CT, MRI, angiography) excluding coronary occlusion (classified as MI) and cerebral artery occlusion (classified as stroke). Arterial Thrombosis: Aortic, Axillary, Humeral, Radial, Iliac, Femoral, Tibial, Celiac Trunk, Mesenteric Arteries. Occurrence of an acute complete deep vein thrombosis within 28 days post‐surgery authenticated by imaging (ultrasound, CT, MRI, scintigraphy, angiography). Note: Peripheral superficial vein thromboses are not counted. Venous Thrombosis: Pulmonary embolism, deep and occlusive venous thrombosis (femoral, iliac, axillary, jugular, and vena cava)

*(iii) stroke:* Occurrence of an acute stroke of ischemic or hemorrhagic origin within 28 days post‐surgery authenticated by imaging (CT, MRI, angiography). Defined by the occurrence of a focal neurological deficit with symptoms lasting more than 24 hours.

*(vi) acute pulmonary oedema:* New or worsening dyspnea and/or hypoxemia associated with imaging evidence of heart failure and increased pulmonary capillary pressure. Improvement with treatment (diuretics, vasodilators, positive pressure ventilation) and absence of other causes explaining the dyspnea support the diagnosis. Note: Cases with detected natriuretic peptide elevation were reviewed by the adjudication committee.

*(v) cardiogenic shock:* Defined by systolic blood pressure less than 90mmHg for more than an hour unresponsive to IV resuscitation and/or heart rate correction and associated with signs of hypoperfusion. May also be defined by a systolic blood pressure less than 90mmHg that increases beyond 90mmHg within an hour with inotropic or vasopressor agents with or without mechanical support.

*(vi) acute severe hypertension:* Episode of hypertension with mean arterial pressure ≥ 130 mmHg requiring intravenous antihypertensive treatment.

*(vii) de novo cardiac arrhythmia requiring therapeutic intervention:* An episode of de novo supraventricular (Atrial Fibrillation/Flutter) or ventricular arrhythmia (Ventricular Tachycardia/Fibrillation) within 28 days postoperatively requiring therapeutic intervention.

| **Variable** | **Data input** |
| --- | --- |
| **General information** |  |
| Hospital site | 1-7 |
| Date of operation | DD/MM/YYYY |
| Age | years |
| Sex | m=0 f=1 |
| ASA classification | 1-4 |
| **Risk factors** |  |
| Diabetes mellitus | n=0 y=1 |
| Heart failure | n=0 y=1 |
| Stroke or transient ischaemic attack | n=0 y=1 |
| Hypertension | n=0 y=1 |
| Ischaemic heart disease | n=0 y=1 |
| Peripheral vascular disease | n=0 y=1 |
| **Surgical procedure category** | 1=Orthopaedic, 2=Gynaecology, 3=Upper GI, 4=Hepato-biliary, 5=Vascular, 6=Urology-Kidney, 7=Lower GI, 8=Other |
| **Cardiac medication** |  |
| ACE-inhibitor | n=0 y=1 |
| ARB | n=0 y=1 |
| Other cardiac medication | n=0 y=1 |
| **Perioperative RAS-inhibitor management** |  |
| Preoperative stop of ACE-inhibitor? | n=0 y=1 n/a=3 |
| Postoperative restart of ACE-inhibitor? | n=0 y=1 n/a=3 |
| When was ACE-inhibitor restarted? | 0= within 12h after surgery, 1=12-24h after surgery, 2=24h after surgery, 3=48h or more after surgery, 4=n/a |
| Preoperative stop of ARB? | n=0 y=1 n/a=3 |
| Postoperative restart of ARB? | n=0 y=1 n/a=3 |
| When was ARB restarted? | 0= within 12h after surgery, 1=12-24h after surgery, 2=24h after surgery, 3=48h or more after surgery, 4=n/a |

**Supplementary Table 2: Data collection tool for national service evaluation**. ACE, Angiotensin-converting enzyme; ARB, Angiotensin receptor blocker; RAS, Renin-angiotensin system; GI, gastrointestinal; ^a^American Society of Anaesthesiology grades are defined as follows (grade 5 patients were not eligible for inclusion): 1, a healthy patient; 2, a patient with mild systemic disease that does not limit physical activity; 3, a patient with severe systemic disease that limits physical activity; and 4, a patient with severe systemic disease that is a constant threat to life.

| Why are we doing this survey? | | |
| --- | --- | --- |
| Your expertise is crucial in improving patient care during major surgeries. We understand from previous research that even minor blood pressure changes can lead to severe myocardial injury, with mortality risks. Patients with existing cardiac conditions often take ACE-inhibitors and ARBs but deciding whether to continue these medications before surgery is complex and can affect outcomes.  The recent clinical trial SPACE, suggests stopping these drugs may increase the risk of myocardial injury, but the impact varies with cardiac disease type. We need your insights to better understand this treatment decision process.  Your survey responses will help refine guidelines, ensuring safer surgery for patients. | | |
| **Clinician Survey Section** | **Question** | **Answer options** |
| Complete if you work in a UK NHS hospital providing care to patients undergoing major surgery. | What is your specialty? | - Surgeon - Anaesthetist - Physician - Other (please specify): |
|  | Which part of perioperative period do you provide patient care for? (tick all that apply) | - Pre-operative assessment clinics and care planning - In hospital before surgery - During surgery - In hospital after surgery |
| How you manage patients scheduled for major non-cardiac surgery who take ACE-inhibitors and ARBs | What advice do you give patients taking ACE-inhibitors who are preparing for major non-cardiac surgery? | - Continue ACE-inhibitor - Stop two or more days before surgery - Stop one day before surgery - Stop on the day of surgery itself - Stop but duration varies with the patient and drug |
|  | If you did stop ACE-inhibitors, when would you normally re-start them after major surgery? (select one option closest to your usual practice) | - Within 12 hours after surgery - 12-24 hours after surgery - 24 hours after surgery - 48 hours or more after surgery |
|  | What advice do you give patients taking ARBs who are preparing for major non-cardiac surgery? | - Continue ARB - Stop two or more days before surgery - Stop one day before surgery - Stop on the day of surgery itself - Stop but duration varies with the patient and drug |
|  | If you did stop ARBs, when would you normally re-start them after major surgery? (select one option closest to your usual practice) | - Within 12 hours after surgery - 12-24 hours after surgery - 24 hours after surgery - 48 hours or more after surgery |
|  | Please rank these goals from 1 to 5, in terms of importance when deciding whether to stop or continue ACE-inhibitors and ARBs before major surgery? | - I want to avoid LOW blood pressure during and after surgery - I want to avoid HIGH blood pressure during and after surgery - I want to avoid heart failure during and after surgery - I want to avoid changing cardiac medications when a patient is stable - I am often uncertain of the best approach |
|  | Does your hospital have routine guidance on stopping or continuing ACE-inhibitors and ARBs before major non-cardiac surgery? | - We have no locally agreed guideline - We have a guideline, but we only use this occasionally - We have a guideline we use in most or all patients |
|  | Is there anything else you would like to tell us about your approach to stopping or continuing cardiac medications before major non-cardiac surgery |  |
|  | If NIHR funded a major trial of stopping vs continuing ACE-inhibitors and ARBs before major non-cardiac surgery, how likely would you be to support randomising most of your patients? We recognise this will not be appropriate for every patient | - Very likely - Likely - Neither likely nor unlikely - Unlikely - Very unlikely |
| A possible clinical trial of stopping versus continuing ACE-inhibitors and ARBs before major non-cardiac surgery | If NIHR funded a major trial of stopping vs continuing ACE-inhibitors and ARBs before surgery, would your hospital be likely to take part? | - Likely - Neither likely nor unlikely - Unlikely - Very unlikely |
|  | Would you be interested in hearing more if the proposed clinical trial is funded? If so, please provide a contact email |  |

**Supplementary Table 3: Clinician questionnaire.** ACE, Angiotensin-converting enzyme; ARB, Angiotensin receptor blocker; UK, United Kingdom; NHS, National Health Service; NIHR, National Institute for Health and Care Research

| **Trial ID** | **Trial title** | **Relevant reasons why the trial was not published** |
| --- | --- | --- |
| NCT04506372 | Management of Angiotensin Inhibitors During the Perioperative Period (AIPOP) | Terminated due to slow recruitment rate (stated in registration) |
| NCT01867047 | ACE-Inhibitor Effects on Total Hip and Knee Arthroplasty Patients | Following an interim analysis the study team felt that there was enough data that showed no significant difference (stated in registration) |
| NCT01091961 | Study of Preoperative Management of Angiotensin Converting Enzyme Inhibitor (ACEi) and Angiotensin Receptor Blocker (ARB) Medications (PASS) | Analysis after completion revealed corruption of the randomisation process by a clinician (contacted principal investigator for clarification) |

**Supplementary Table 4: Unpublished trials.** ACE, Angiotensin-converting enzyme; ARB, Angiotensin receptor blocker

| **Patient baseline and operative characteristics** | **Number of patients with available data – N (%)** |
| --- | --- |
| *Sex* |  |
| Male | 145 (45.9) |
| Female | 171 (54.1) |
| *Age (years)* |  |
| Range (min-max) | 50 - 92 |
| *^a^ American Society of Anaesthesiology grade* |  |
| I | 0 (0) |
| II | 120 (38.0) |
| III | 188 (59.5) |
| IV | 8 (2.5) |
| *^b^ Risk factors* |  |
| Diabetes mellitus | 122 (38.6) |
| Heart failure | 31 (9.8) |
| Ischaemic heart disease | 78 (24.7) |
| Stroke or transient ischaemic attack | 25 (8.0) |
| Peripheral vascular disease | 24 (7.6) |
| Hypertension | 302 (95.6) |
| *Cardiac medication* |  |
| RAS-inhibitors* |  |
| ACE-Inhibitor | 210 (66.5) |
| ARB | 107 (33.9) |
| Other cardiovascular medication | 220 (69.6) |
| *Surgical procedure category* |  |
| Orthopaedic | 57 (18.0) |
| Vascular | 23 (7.3) |
| Gynaecology | 17 (5.4) |
| Urology and kidney | 17 (5.4) |
| Upper gastro-intestinal | 21 (6.6) |
| Lower gastro-intestinal | 47 (14.9) |
| Hepato-biliary | 29 (9.2) |
| Other | 105 (33.2) |

**Supplementary Table 5: Patient baseline and operative characteristics from national service evaluation.** SD, standard deviation; IQR, Interquartile range; ACE, Angiotensin-converting enzyme; ARB Angiotensin receptor blocker; ^a^ American Society of Anaesthesiology grades are defined as follows (grade 5 patients were not eligible for inclusion): 1, a healthy patient; 2, a patient with mild systemic disease that does not limit physical activity; 3, a patient with severe systemic disease that limits physical activity; and 4, a patient with severe systemic disease that is a constant threat to life. ^b^ Patient may have more than one risk factor. * One patient received both ACE-inhibitor and ARB.

| **Preoperative RAS management by surgical specialty** | | | |
| --- | --- | --- | --- |
|  | **Patients with any RAS-inhibitor**  **Number of patients with available data – no. (%), N= 316 (100)** | | |
| **Surgical specialty** | **all** | **paused** | **continued** |
| Orthopaedic | 57 (100) | 50 (87.7) | 7 (12.3) |
| Vascular | 23 (100) | 16 (69.6) | 7 (30.4) |
| Gynaecology | 17 (100) | 13 (76.5) | 4 (23.5) |
| Urology and renal | 17 (100) | 11 (64.7) | 6 (35.3) |
| Upper gastro-intestinal | 21 (100) | 13 (61.9) | 8 (38.1) |
| Lower gastro-intestinal | 47 (100) | 31(66.0) | 16 (34.0) |
| Hepato-biliary | 29 (100) | 20 (69.0) | 9 (3.10) |
| Other | 105 (100) | 94 (89.5) | 11 (10.5) |

**Supplementary Table 6: Preoperative management of RAS inhibition by surgical specialty**

| **Preoperative RAS management by risk factor** | | | |
| --- | --- | --- | --- |
|  | **Patients with any RAS-inhibitor**  **Number of patients with available data – no. (%), N= 316 (100)** | | |
| **Risk factor** | **all** | **paused** | **continued** |
| Diabetes mellitus | 122 (100) | 96 (78.7) | 26 (21.3) |
| Heart failure | 31 (100) | 20 (64.5) | 11 (35.5) |
| Ischaemic heart disease | 78 (100) | 54 (69.2) | 24 (30.8) |
| Stroke or transient ischaemic attack | 25 (100) | 20 (80.0) | 5 (20.0) |
| Peripheral vascular disease | 24 (100) | 18 (75.0) | 6 (25.0) |
| Hypertension | 302 (100) | 239 (79.1) | 63 (20.9) |

**Supplementary Table 7: Preoperative management of RAS inhibition by risk factor**
